# Interpreting insect behavior through the lens of executive functions

**DOI:** 10.3389/fnbeh.2025.1638374

**Published:** 2025-08-04

**Authors:** Bartosz Baran, Michał Obidziński, Mateusz Hohol

**Affiliations:** Mathematical Cognition and Learning Lab, Copernicus Center for Interdisciplinary Studies, Jagiellonian University, Krakow, Poland

**Keywords:** executive functions, insect cognition, mushroom bodies, central complex, behavioral flexibility, behavioral control, comparative neuroethology

## Abstract

Despite miniature brains, insects exhibit flexible, adaptive, and goal-directed responses. Behaviors indicating rule abstraction and complex decision-making challenge the long-standing view of insects as rigid organisms limited to fixed reflexes. Here, we propose a new perspective: interpreting insect behavior through the lens of executive functions (EF). EF refers to a set of cognitive processes enabling behavioral control in situations requiring goal-directed action or adaptation to demanding conditions. Central among EF are inhibition (suppressing automatic, task-irrelevant responses), shifting (switching between strategies or rules), and updating (maintaining and revising relevant information), yet working memory, attention, planning, decision-making, and metacognition are also related to a widely understood set of EF. We argue that insect cognition can be productively reconsidered using the EF framework. Many behaviors documented in the literature align with EF components, even if not explicitly labeled as such. Others can be reinterpreted as EF-driven. Importantly, we show that EF-based interpretations support testable predictions: if executive control is involved, behavior should follow developmental trajectories, exhibit trade-offs between speed and accuracy, and adapt to changing contexts–patterns not expected from fixed heuristics or reflexes. Nonetheless, applying EF concepts to insects comes with challenges. Standard EF paradigms were originally developed to test human participants and often rely on language and explicit task instructions. Moreover, superficially flexible behaviors may still result from specialized, domain-specific routines rather than general cognitive control. Nevertheless, when used carefully, the EF perspective provides a structured, functional framework for studying insect cognition, enabling precise comparison across species with well-established concepts.

## 1 Introduction

Over the two decades, a growing body of evidence has revealed the previously unanticipated complexity of insect behavior and cognition (Döring and Chittka, 2011). Studies focusing predominantly on species such as bees (Giurfa, 2007), wasps (Weise et al., 2022), ants (Czaczkes, 2022), but also fruit flies (Grover et al., 2022), crickets (Baran et al., 2023), and locusts (Ben-Nun et al., 2013), have demonstrated a broad range of complex capacities, including associative and observational learning, decision-making, rule generalization, and even the abstraction of symbolic information. Such findings challenge long-standing views that insect behavior is predominantly reflexive, rigid, and governed solely by stimulus-response mechanisms (Giurfa, 2015). Insects, despite their comparatively diminutive nervous systems, exhibit cognitive and behavioral capacities that seem to parallel those observed in animals with larger brains (Perry et al., 2017).

This raises a foundational question: How should these capacities be accounted for? Are they a product of internal control systems akin to those supporting higher cognition in vertebrates, or do they emerge from highly domain-specific associative learning or from sets of heuristics precisely fine-tuned over evolutionary history? Current interpretations are often polarized. Those inclined toward reductive explanations point out the danger of falling for undue anthropomorphic projections (Dhein, 2023). At the same time, their opponents raise the issue of a lack of evolutionary justification for excessive reductionism (Hohol et al., 2017a). Indeed, insect neuroanatomy and neurophysiology is immensely dissimilar to the vertebrate one, yet conversely, in theory, arbitrary complex behavior may be reduced to a sufficiently extensive set of simpler actions (Skinner, 1938). Nevertheless, there seems to be a lack of a coherent framework facilitating the formulation of hypotheses, the testing of which could help resolve these discussions.

Executive Functions (EF), a set of domain-general cognitive processes that enable flexible, goal-directed behavior and regulate internal states in response to environmental demands (Diamond, 2013; Cristofori et al., 2019), arise as a promising candidate for application to insects’ behavioral neuroscience. EF are theorized to occupy a high-level regulatory role within the cognitive system, orchestrating lower-level processes to enable adaptive and context-sensitive behavior across changing environmental demands. Although the concept of EF emerged within human neuropsychology and is strongly associated with the function of the prefrontal cortex (PFC), recent comparative studies indicate that analogous, EF-like capabilities arise in vertebrates such as birds independently of cortical structures (Emery and Clayton, 2004). This suggests the potential of EF as a framework for describing cognitive processes on a more abstract level and as an organizing principle for understanding adaptive behavior across contexts, while nonetheless prompting questions about convergence and/or functional homology of anatomically differing structures.

This paper aims to position the EF framework as a promising perspective on insect behavior and cognition, entailing a distinctive set of characteristics that could be identified and tested, thus facilitating the development of conceptual and methodological tools for better exploring insects’ cognitive complexity.

First, we outline the EF framework’s origins in human psychology and neuroscience, followed by its adaptation to non-human animals. Subsequently, we move to insect cognition by reviewing cases where insect behavior has already been explicitly described in EF-related terms, and, most importantly, applying the framework *de novo* to reinterpret findings from studies that did not originally invoke EF-related concepts. While we emphasize the functional over structural characteristics of EF, we also consider potential neural substrates in insects that may support EF-like processes. Finally, we discuss the strengths and limitations of this perspective.

## 2 EF in human neuropsychology

Executive functions are usually conceptualized as a set of processes at the top of the cognitive hierarchy (“metacognitive”) and are responsible for behavioral control, planning, problem-solving, achieving goals, and adapting to situational demands (Diamond, 2013; Cristofori et al., 2019). They are closely related to working memory (WM), attention, and cognitive flexibility. They are domain-general processes that influence all forms of cognition when control is required. EF impairments lead to deficits in non-automatic cognitive processes and behaviors. In humans, healthy development and effective performance of EF (Thompson and Steinbeis, 2020) are robust predictors of life outcomes, crucial for adaptation and decision-making (Moffitt et al., 2011). EF typically emerge in early childhood, strengthen through adolescence, peak in early adulthood, and decline with age, though not uniformly across all components (Lacreuse et al., 2020; Ferguson et al., 2021).

The EF concept is strongly linked to the clinical context, particularly to the neuropsychological perspectives on PFC dysfunction (Friedman and Robbins, 2022); however, the cybernetic tradition has played an important role in its development (Miller et al., 1968; Baddeley, 2010). The term EF is, to some extent, used interchangeably with cognitive control. Despite a well-established grounding in the PFC, EF are also associated with other anterior and posterior cortical regions (Bettcher et al., 2016).

Despite several categorizations of EF, one of the most widely used is a three-component model, comprising inhibition, shifting, and updating by Miyake et al. (2000). Inhibition refers to the process of suppressing an automatic reaction to a stimulus and/or suppressing task-irrelevant perceptions. Shifting refers to the process of exerting controlled changes between different tasks. Finally, updating refers to the process of keeping task-relevant information in one’s WM. Notably, EF categories sometimes overlap, reflecting varying research approaches and diverse terminology. In addition to just mentioned, the following concepts can be listed as examples of EF: cognitive flexibility, behavioral control, planning, decision-making, and problem-solving (Miyake et al., 2000; Cristofori et al., 2019).

## 3 Cross-species application of EF

Psychological concepts are often applied to research on other animals (Smith et al., 2003). Typically, such an application enables researchers to test the evolutionary roots of cognitive mechanisms and examine how shared or divergent neural architectures support similar functions (de Waal and Ferrari, 2010). This is exactly what happened in the case of research on EF in non-human primates, which focused on homologies with human PFC functions (Mansouri et al., 2017; Lacreuse et al., 2020). Over time, research expanded to include other mammals, notably domestic dogs, whose co-evolution with humans makes them valuable models for studying cognitive control in ecologically relevant contexts (Foraita et al., 2022). Parallel to these developments, rodents became key, though indirect, models in EF research due to their translational relevance for modeling human cognitive disorders (Bizon et al., 2012).

As exemplified by avian cognition research, EF concepts can be fruitfully applied to non-mammalian species. Corvids and psittacines–birds known for their broad behavioral repertoires and complex social interactions–have consistently shown proficiency in tasks probing EFs (Bobrowicz and Greiff, 2022). Crow species and African gray parrots, for instance, demonstrate advanced inhibitory control in detour-reaching tasks and delayed-rewards exchanges, as well as robust WM in object permanence and transposition paradigms (Hoffmann et al., 2011; Rössler and Auersperg, 2023). Reversal learning and multi-access puzzle tasks, often interpreted as indicators of cognitive flexibility, underscore the control capabilities of these taxa (Auersperg et al., 2011). Such findings challenge the centrality of cortical substrates to EF, as birds achieve functionally comparable outcomes, relying on pallial regions, including the nidopallium caudolaterale, which lacks the laminar organization of the mammalian neocortex yet seemingly supports comparable integrative processing (Emery and Clayton, 2004; Rose and Colombo, 2005; Kersten et al., 2024). This neuroanatomical divergence, coupled with functional convergence or homology, strengthens the case for defining EF not by structural correlates but by computational characteristics.

These findings have prompted inquiries into EF-like capacities across vertebrates, including species not typically characterized by large brains. Fish species such as guppies, cleaner wrasses, and zebrafish (Parker et al., 2013) have shown evidence of engaging in behaviors suggestive of EF. Guppies, for example, have succeeded in detour tasks requiring inhibition and spatial flexibility (Lucon-Xiccato et al., 2017; Gatto et al., 2018), while cleaner wrasses have demonstrated the ability to modulate their behavior based on the perceived perspective of their social partners (McAuliffe et al., 2021), indicating context-sensitive decision-making. Among recent findings are demonstrations of EF-like behavior in invertebrates, namely, cephalopod mollusks, particularly octopuses and cuttlefish. Despite their divergent neuroanatomy, cephalopods have shown advanced control and learning flexibility (Jozet-Alves et al., 2013). Cuttlefish, for example, have been shown to forgo an immediate but less-preferred rewards in favor of a delayed, higher-value option–a clear functional parallel to human delay-of-gratification paradigms (Schnell et al., 2021b). These behaviors have been further correlated with performance in reversal learning paradigms, suggesting an integrated system of inhibitory control and flexibility. Similarly, octopuses have demonstrated the ability to adapt to changing problem-solving contexts and to remember prior outcomes in decision tasks (Schnell et al., 2021a). These findings are especially significant in the discussed context, given the absence of any directly homologous structures to the vertebrate cortex.

## 4 Executive functions in insects

Against the background just described, we propose taking the next step–applying the EF concepts to insects, whose neural architecture is extremely miniaturized. Our proposal is not made in a vacuum and may not appear entirely original, as several insect studies have already used terms associated with EF. Thus, in Section “4.1 Verbatim usage,” we briefly review the verbatim use of EF-related terminology, focusing primarily on studies involving bees and ants. While these studies provide a credible starting point, some of them do not situate individual terms within the broader framework of EF. This is evident in the lack of cross-referencing between specific terms (e.g., the absence of any indication that inhibition and updating may reflect distinct yet related capacities), the omission of references to foundational authors or key publications on EF, and–notably–the absence of the term “executive functions” itself. These limitations are important, as the terms used in the existing literature do not necessarily denote EF as typically understood in neuropsychology. Before proposing means to clarify whether a particular capacity in insects sufficiently resembles EF in vertebrates to warrant such a label, in Section “4.2 Functional inference” we draw attention to a somewhat reversed situation–namely, cases in which certain processes are not explicitly labeled as EF, even when their executive nature could be readily inferred. Next, in Section “4.3 Conceptual integration,” we describe another, more interpretively challenging context in which EF may be involved in the execution of cognitive tasks, for example, as is the case of tasks designed to assess numerosity processing, where an animal must inhibit conflicting perceptual cues in favor of numerical information.

### 4.1 Verbatim usage

Executive functions-related terminology has already been used in several studies on insect cognition (Table 1). For instance, Giurfa’s (2007) comprehensive review uses several core EF terms verbatim to account for complex cognitive performance in honeybees. For example, *problem solving* is introduced in the context of non-elemental forms of associative learning, where bees must resolve ambiguity in stimulus-rewards contingencies–such as in negative patterning and biconditional discrimination tasks–by treating compound stimuli as more than the sum of their parts. This capacity, Giurfa argues, reflects flexible computation, and is presented as evidence of sophisticated problem-solving. *Inhibition* is discussed in relation to side-specific olfactory conditioning, in which bees are trained to associate the same odorant with opposing outcomes depending on which antenna is stimulated. Successful performance requires not only learning new associations but also actively suppressing context-inappropriate responses, highlighting inhibition as an operational requirement for task execution. Finally, *working memory* is addressed in the analysis of delayed matching-to-sample paradigms, where bees must retain a sample stimulus in memory across a temporal delay before selecting a matching or non-matching option. Giurfa provides evidence that this retention spans approximately 5 seconds, directly linking performance to the maintenance component of WM.

**TABLE 1:** Verbatim usage of executive function (EF)-related terminology in insect cognition studies. Each entry lists the EF term, source, species studied, and a brief description of the behavioral context.

References	Article title	Species studied	Usage context
**Inhibition**			
Giurfa (2007)	Behavioral and neural analysis of associative learning in the honeybee: a taste from the magic well	*Apis mellifera* (honeybee)	Suppression of innate responses during conditioning; evidence from proboscis extension reflex studies
Chittka and Niven (2009)	Are bigger brains better?	Various species (e.g., *Apis mellifera*, *Drosophila melanogaster*)	Discussion of neural efficiency and behavioral restraint related to brain size and control
Buatois et al. (2020)	Higher-order discrimination learning by honeybees in a virtual environment	*Apis mellifera* (honeybee)	Inhibition in higher-order learning tasks in a virtual maze environment
Czaczkes et al. (2022)	Conflict interference in an insect	*Lasius niger (black garden ant)*	Conflict inference in the stroop-like task paradigm
Gibbons et al. (2022)	Descending control of nociception in insects?	*Bombus terrestris* (bumblebee)	Central control over nociceptive (pain-like) responses under motivational conflict
**Shifting**			
Perry and Barron (2013)	Honeybees selectively avoid difficult choices	*Apis mellifera* (honeybee)	Avoidance of difficult decisions; context-based rule shifting
MaBouDi et al. (2020)	Bumblebees learn a relational rule but switch to a win-stay/lose-switch heuristic after extensive training	*Bombus terrestris* (bumblebee)	Shift from relational rule learning to win-stay/lose-switch heuristic under extended training
**Updating**			
Buatois et al. (2020)	Higher-order discrimination learning by honeybees in a virtual environment	*Apis mellifera* (honeybee)	Learning and revising complex discrimination tasks using updated information in a VR setup
Adam et al. (2022)	Fast Learners: one trial olfactory learning in insects	Various species (e.g., *Drosophila melanogaster*, *Cataglyphis fortis*, *Apis mellifera*)	One-trial olfactory learning suggests rapid memory updating capabilities
**Working memory**			
Giurfa (2007)	Behavioral and neural analysis of associative learning in the honeybee: a taste from the magic well	*Apis mellifera* (honeybee)	Associative learning mechanisms requiring short-term memory
Avarguès-Weber and Giurfa (2013)	Conceptual learning by miniature brains	*Apis mellifera* (honeybee)	Abstract concept learning relying on temporary memory retention
Avarguès-Weber et al. (2014)	Conceptualization of relative size by honeybees	*Apis mellifera* (honeybee)	Relative size judgment indicating use of visual working memory
Sherry and Strang (2015)	Contrasting styles in cognition and behavior in bumblebees and honeybees	*Bombus spp*. (bumblebee), *Apis mellifera* (honeybee)	Comparative analysis of memory use in different bee species
Baran et al. (2023)	Geometry-based navigation in the dark: layout symmetry facilitates spatial learning in the house cricket	*Acheta domesticus* (house cricket)	Geometry-based navigation relying on memory of spatial layouts
Czaczkes (2022)	Advanced cognition in ants	Various ant species (e.g., *Aphaenogaster subterranea, Paratrechina longicornis*)	Goal maintenance across spatially displaced tool use sequence
Martín-Ordás (2022)	Frames of reference in small-scale spatial tasks in wild bumblebees	*Bombus spp.* (bumblebee)	Spatial task completion requiring short-term memory of object location
**Cognitive flexibility**			
Loukola et al. (2017)	Bumblebees show cognitive flexibility by improving on an observed complex behavior	*Bombus terrestris* (bumblebee)	Improvement and modification of an observed behavior (puzzle solving)
MaBouDi et al. (2020)	Bumblebees learn a relational rule but switch to a win-stay/lose-switch heuristic after extensive training	*Bombus terrestris* (bumblebee)	Behavioral strategy change from abstract to simple heuristics over training
**Problem solving**			
Giurfa (2007)	Behavioral and neural analysis of associative learning in the honeybee: a taste from the magic well	*Apis mellifera* (honeybee)	Associative and configural learning used to solve conditioned tasks
Simons and Tibbetts (2019)	Insects as models for studying the evolution of animal cognition	Various species (e.g., *Polistes fuscatus, Polistes metricus*)	Review of insect models showing evolutionary diversity of problem-solving abilities
**Planning**			
Avarguès-Weber and Giurfa (2013)	Conceptual learning by miniature brains	*Apis mellifera* (honeybee)	Discussion of higher-order cognition involving sequence planning
Chittka et al. (2019)	Editorial: the mechanisms of insect cognition	Various species (e.g., *Bombus terrestris, Periplaneta americana*)	Editorial overview linking advanced behaviors like trap lining to planning
Czaczkes (2022)	Advanced cognition in ants	Various ant species (e.g., *Aphaenogaster subterranea*, *Aphaenogaster senilis*)	Multi-step foraging behavior involving sub-goal execution toward delayed rewards
**Decision-making**			
Avarguès-Weber and Giurfa (2013)	Conceptual learning by miniature brains	*Apis mellifera* (honeybee)	Linking rule learning with value-based decision processes
Barron et al. (2015)	Decision-making and action selection in insects: inspiration from vertebrate-based theories	General insect models (e.g., *Apis mellifera*, *Drosophila melanogaster*)	Modeling insect behavior using vertebrate-like decision networks
**Behavioral control**			
Moore (2001)	Honey bee circadian clocks: behavioral control from individual workers to whole-colony rhythms	*Apis mellifera* (honeybee)	Circadian rhythms coordinating individual and colony-level behavior
Cheng et al. (2002)	Self-control in honeybees	*Apis mellifera* (honeybee)	Self-control demonstrated through delayed gratification tasks
Giurfa (2007)	Behavioral and neural analysis of associative learning in the honeybee: a taste from the magic well	*Apis mellifera* (honeybee)	Conditioned learning with inhibitory and motivational regulation

Next, Czaczkes (2022) review of ant cognition includes several EF-related terms used explicitly. The term *working memory* is used to explain tool-use behaviors in which ants suppress feeding, search for an appropriate tool, and later return to the food source–thus requiring them to maintain the goal location in memory across spatially and temporally displaced sub-goals. This sequence reflects the core executive demand of retaining and manipulating task-relevant information during ongoing action. Similarly, *planning* is invoked to describe the coordination of such multi-step behaviors, where ants must construct a representation of the current state, identify a goal, and organize intermediate steps to achieve it.

While these instances demonstrate that EF-related terms are present in the literature in a direct and structured form, relevant behaviors are also described in functional terms that strongly align with EF constructs, but do not use more specific terms. For example, Gibbons et al. (2022) report a “*centrally controlled* reduction of nocifensive behavior” (p.1) in bumblebees–an instance that maps closely onto inhibition, as it involves the suppression of an innate, reflexive response under context-dependent conditions.

Similar cases could be found in Czaczkes (2022) review, which dedicates a section to *cognitive control*, defined explicitly as “the repression of an instinctive, preferred, or dominant response in favor of a more appropriate learned response when the two responses conflict” (p.54). This definition directly mirrors inhibitory control, indeed, it is even illustrated via reference to the Stroop task (Czaczkes et al., 2022). Furthermore, Czaczkes (2022) review invokes the notion of executive *control* as part of an ongoing debate on whether such control is required to explain suppression of dominant responses. While the term is not elaborated in detail, its appearance suggests a significant conceptual alignment with the EF framework.

Taken together, EF-related terminology is present in the insect cognition literature, encompassing virtually all core components of executive function. However, these terms are typically employed in isolation.

### 4.2 Functional inference

A somewhat opposite situation occurs when EF components are neither explicitly named nor described, yet can be inferred from the experimental design and observed behavior. For example, in cricket studies involving a dry analog of Morris Water Maze (Wessnitzer et al., 2008; Baran et al., 2023), in which animals had to locate a cool spot within an arena whose floor was heated to a uniformly noxious temperature, success demanded the suppression of an innate reflex. Crickets, especially when exposed to stress, exhibit a drive to remain in close contact with surfaces–thigmotactic, shelter-seeking escape behavior–while the contextually appropriate spatial strategy required exploration and occupation of the centers of the arenas. Performance improvements over trials and trajectory analyses showing a transition from wall-following provide evidence for the suppression of a reflexive response, which can be interpreted as inhibition. A similar case can be found in honeybees, which can be conditioned to produce an aversive sting extension reflex in response to innately attractive pheromonal or floral odors after training in which these odors were paired with a noxious stimulus (Roussel et al., 2012). In this paradigm, bees override strong innate approach tendencies and instead exhibit learned aversive behavior, demonstrating a reversal of hardwired response valence, which also fits the inhibition.

### 4.3 Conceptual integration

Yet another situation arises when EF can be identified as the processes underlying complex skills. Here, the presence of EF is inferred based on the assumption that if cognitive processes known to be EF-dependent exhibit consistent behavioral patterns across species, then similar patterns observed in insects may likewise imply EF-like mechanisms. For example, studies on spatial-numerical associations (SNAs) demonstrate that bees associate smaller numerosities with the left and larger ones with the right side of space (Giurfa et al., 2022; Kuo et al., 2025), similarly to vertebrates (Vallortigara, 2018). While SNAs cannot be explained solely by referring to EF, they can be understood as phenomena that, in addition to basic numerical perception, also recruit EF in non-trivial ways to function effectively. This claim is supported by findings indicating that inhibitory control plays an important role in human SNAs (Wood et al., 2008; Zhang et al., 2024). If insect numeracy shares properties with that of vertebrates, comparable mechanisms may also be involved.

More generally, as argued by Hohol et al. (2017b), performance in non-symbolic numerosity perception (e.g., dot patterns) depends on the ability to inhibit conflicting perceptual cues (e.g., area, density, contour length) in favor of numerical information. Without such inhibition, task performance is confounded by irrelevant stimulus dimensions, impairing numerosity discrimination. Notably, the honeybee studies on numerosity perception also used dot patterns (Howard et al., 2018, 2022; Giurfa et al., 2022). Furthermore, honeybees also exhibit conflict effects, such as performance deterioration under reverse training or a reversion to default mappings during extinction, that closely mirror Stroop-like congruency effects, which in vertebrates are known to depend on inhibitory control (Lauwereyns et al., 2000). Thus, if insects’ numerical cognition is prone to conflict effects, and if successful task performance requires the suppression of conflicting cues, this may imply the presence of *inhibition*.

A similar interpretation could be applied to *shifting* (e.g., rule reversal or behavioral reconfiguration) and *updating* (e.g., conceptual generalization or memory-dependent navigation). For example, shifting may underlie insects’ behavior in tasks that require switching between different rules or actions to achieve a goal (Loukola et al., 2024). As shifting in humans and other animals refers to the ability to perform in such a manner, the observation of this behavior in insects suggests, at the very least, a similar mechanism in their cognitive architecture. This is especially important, as the demands of the observed performance were analogous to those of tasks used in both clinical settings to assess set-shifting function (Rabinovici et al., 2015), and experimental settings examining or manipulating shifting performance (Purić and Pavlović, 2012; Nieznański et al., 2015) in humans. Finally, updating of WM is a candidate cognitive mechanism underlying insect behaviors that appear to rely on previously available information, rules, and even prospective planning (Giurfa, 2007; Czaczkes, 2022).

## 5 Executive functions vs. complex cognition

To avoid turning EF into a catch-all label for complex cognition in insects, it is important to identify cognitive phenomena that, while complex, are not executive functions themselves. Following Knauff and Wolf (2010), by complex cognition we mean that which “takes place under complex conditions in which a multitude of cognitive processes interact with one another or with other non-cognitive processes” (p. 100). Therefore, phenomena such as reasoning, or knowledge use and transfer are examples of complex cognition, but they are not EFs themselves (even if there is a possibility of interaction between them). Here, we briefly present a handful of examples of complex cognition in insects that should not be conflated with EF.

Ants have been observed to learn new object affordances and to act according to acquired knowledge (Poissonnier et al., 2023). This exemplifies a complex cognition, constituted by interactions among perception, memory, and social learning, that might interact with EF (e.g., inhibition might be needed to stop an initial reaction), but it is not EF itself. Next, Ebina and Mizunami (2020) found that crickets prefer food sources where a living conspecific had been encountered and avoid those associated with dead conspecifics. This presents a form of reasoning: “If a conspecific is alive, then the food is safe; if it is dead, the food is dangerous.” However, EF processes are not required for this behavior to occur. Finally, in a study by Solvi et al. (2020), bumblebees performed well in a task requiring cross-modal (in this case, visual and tactile) object recognition. The insects were able to recognize objects presented in a different sensory modality from the one used during the learning phase. This ability represents a form of object abstraction, but does not imply the involvement of EF.

## 6 Candidate neural substrates for EF in insects

Although EFs do not necessarily depend on the presence of a cerebral cortex, the successful application of the EF framework requires identifying the underlying neural substrate.

Two insect brain structures consistently emerge in the context of complex cognition: the mushroom bodies (MBs) and the central complex (CX) (Heisenberg, 2003; Pfeiffer and Homberg, 2014; Barron and Klein, 2016). MBs are bilaterally symmetrical structures crucial for associative learning, memory formation, and sensory integration (Heisenberg, 2003; Farris, 2011). Lesion and imaging studies have shown that MBs are indispensable for tasks requiring the formation of stimulus–rewards contingencies and behavioral flexibility (Zars, 2000; Menzel, 2012). Importantly, the MBs receive multimodal sensory input and contribute to state-dependent behavioral modulation–characteristics reminiscent of the integrative role played by the PFC in vertebrates (Strausfeld et al., 1998; Barron and Klein, 2016).

Central complex, on the other hand, is a singular structure located in the midline of the insect brain, involved in spatial orientation, motor control, and decision-making (Strausfeld and Hirth, 2013; Pfeiffer and Homberg, 2014; Honkanen et al., 2019). It appears particularly important for action selection and goal-directed navigation, functioning through the integration of internal states and external cues to orchestrate context-appropriate responses (Turner-Evans and Jayaraman, 2016; Honkanen et al., 2019; Goulard et al., 2023). In the fruit fly, CX integrates internal variables such as sleep homeostasis (Flores-Valle et al., 2021) and path-integrated heading direction to modulate navigational decisions, demonstrating internal control over spatially guided actions (Seelig and Jayaraman, 2015; Kottler et al., 2019).

These findings suggest that the MBs and CX form part of a distributed control architecture in which executive-like functions may emerge (Menzel, 2012; Pfeiffer and Homberg, 2014; Collett and Collett, 2018).

## 7 Discussion

We propose that several insect behaviors can be systematically interpreted through the EF framework–an approach commonly used in vertebrate cognitive and behavioral neuroscience research. The broader application of the EF framework can help advance insect cognition research in numerous ways. First, it provides a standardized vocabulary for identifying and categorizing behavior dependent on inhibition, WM, and cognitive flexibility–capacities already investigated across a wide range of animals, thus facilitating cross-species comparisons. Next, since many of these concepts have already been used in the insect cognition literature–albeit often without a broader theoretical context–adopting the EF framework would allow for both retrospective reinterpretation of existing findings and unification of terminology in future research in an otherwise fragmented field.

Furthermore, adopting the EF framework opens new research avenues by highlighting developmental patterns and constraints that are characteristic of executive control across taxa (Lacreuse et al., 2020). If insects indeed exhibit EF-like capacities, comparable trajectories, though likely compressed and mechanistically distinct from the vertebrate ones, may be observable. As previously proposed, if the MBs and CX serve as primary substrates for executive-like functions, then it would be expected that developmental changes in their structure could correlate with corresponding shifts in EF-related behavior (Strausfeld and Hirth, 2013; Collett and Collett, 2018). When examining potential trajectories, a sharp distinction must be drawn between hemimetabolous and holometabolous species, due to fundamental differences in their neural ontogeny. In hemimetabolous insects, MBs are known to exhibit incremental development across successive juvenile instars (Farris, 2011; Strausfeld and Hirth, 2013), raising the possibility of a more vertebrate-like pattern of gradual maturation in executive capacities. However, these species remain behaviorally understudied in this context, and systematic links between MB development and EF-like behavior are currently lacking. In contrast, holometabolous insects undergo extensive neural reorganization during metamorphosis, rapidly achieving mature-like neural structures post-eclosion. In these species, subsequent plasticity arises primarily from environmental modulation, most notably, experience-dependent expansion of the MB neuropil, as observed in honeybees transitioning from in-nest tasks to foraging (Menzel, 2012; Collett and Collett, 2018). While this indicates functional refinement post-emergence, it does not imply a prolonged developmental arc akin to the vertebrate prefrontal cortex. Nevertheless, some insect species do exhibit patterns suggestive of developmental and age-related modulation of EF-like behavior, such as cognitive decline in older individuals (Münch et al., 2013), which bears significant resemblance to vertebrate EF dynamics.

Moreover, EF tasks often reveal inherent trade-offs between speed and accuracy, context-dependent modulation of behavior, and limitations in attentional or memory resources–features that distinguish executive processing from more rigid forms of associative learning (Ye et al., 2019; Ibbotson, 2023). Recognizing these hallmarks provides a basis for designing behavioral paradigms that probe not only whether insects can perform a task, but also how their performance varies with internal states, task demands, and conflicting goals. Such refinement may enable deeper cross-species comparisons, including parallel task designs for insects and vertebrates.

However, applying the EF framework to insects entails significant challenges. Notably, numerous EF paradigms used in humans rely on verbal instruction, explicit goal comprehension, and introspective reporting–none of which are accessible in studies involving insect models. EF-related terminology can be easily overextended. Caution is especially important, given that superficially similar outcomes can be driven by distinct cognitive processes (Gatto et al., 2023). Thus, interpreting insect behavior in terms of EF is challenging, particularly when attempting to distinguish between genuinely flexible processes and highly specialized, context-dependent associative mechanisms (Strelevitz et al., 2024). Behaviors that appear adaptive or strategic may, in fact, emerge from domain-specific heuristics rather than from executive functioning.

The EF framework should not become a catch-all label for complex cognition. Its strength lies in its ability to differentiate latent control processes based on characteristic signatures, not merely on specific behavioral outcomes. Despite its limitations, the EF framework offers a powerful comparative tool, encouraging integrative research across behavior, neurobiology, and modeling.

## Data Availability

The original contributions presented in this study are included in this article/supplementary material, further inquiries can be directed to the corresponding authors.
